# The complete mitochondrial genome of the parasitic sheep ked *Melophagus ovinus* (Diptera: Hippoboscidae)

**DOI:** 10.1080/23802359.2017.1347832

**Published:** 2017-07-26

**Authors:** Zhi-Qiang Liu, Nuer Kuermanali, Zhao Li, Shi-Jun Chen, Yuan-Zhi Wang, Han Tao, Chuang-Fu Chen

**Affiliations:** a Institute of Veterinary Medicine, Xinjiang Academy of Animal Science, Urumqi, China;; b College of Animal Science and Technology, Shihezi University, Shihezi, Xinjiang Uygur Autonomous Region, China;; c School of Medicine, Shihezi University, Shihezi, Xinjiang Uygur Autonomous Region, China

**Keywords:** Calyptratae, Hippoboscoidea, phylogenomics, paraphyly, compositional bias

## Abstract

The complete mitochondrial genome (15,573 bp) of an understudied sheep parasite *Melophagus ovinus* was sequenced and characterized. Its organization and characteristics, including the size, structure, gene order, start/stop codon usage and gene overlaps, are largely typical for Diptera. It exhibits very high A + T bias (81%). Posterior probability values in the inferred phylogenetic dendrogram were very high, but Oestroidea and Muscoidea superfamilies were both paraphyletic. The sequence was nested within the Oestridae clade, thus also rendering the family paraphyletic. A larger number of Hippoboscoidea mitogenomes will have to be available to achieve a better phylogenetic resolution.

Until the introduction of insecticides, a globally distributed wingless fly, sheep ked *Melophagus ovinus* (Hippoboscidae), was one of the most damaging ectoparasites of sheep (Lloyd 2002). Recently, the rise of organic farming has allowed its resurgence (Small 2005; Martinković et al. 2012), but as a result of this prolonged period of lesser veterinary relevance, its genetics is very poorly understood, with only 16 sequences of five partial genes deposited in the GenBank (May 2017). Furthermore, as the entire Hippoboscoidea superfamily (Diptera: Calyptratae) currently remains non-represented in terms of complete sequenced mitogenomes, this hinders the progress of the understanding of dipteran evolution (Nelson et al. 2012). To address this, we have sequenced its complete mitogenome.

Sheep ked were collected in the spring of 2014 from the Qinggil County (89°47’–91°04’ E, 45°00’–47°20’N), Xinjiang UAR, China. Specimens were morphologically identified as described before (Lloyd 2002) and genetically by *Cox1* barcoding using BOLD database (Ratnasingham and Hebert 2013): *M. ovinus* was top hit with almost perfect (99.57%) similarity, followed by *Phytoliriomyza melampyga* at 89.54%. Voucher specimens are permanently stored in the Xinjiang Academy of Animal Sciences, Institute of Veterinary Medicine, Laboratory for Parasitic Diseases in Animal Husbandry, 2nd floor, room No. 223, ultra-low temperature (−80°C) refrigerator no. 2, top 1–1 lattice, under the accession number qhc-2014-4. Backup samples are stored under the same number in the basement sample storage room 002, liquid nitrogen tank no. jsc-3. Mitogenome (accession number: KX870852) was amplified, sequenced and annotated as described before (Wen et al. 2017). Supplementary file containing the barcoding results, primer information, detailed information about the studied mitogenome and comparison of the studied Calyptratae mitogenomes is available from a public data repository (https://doi.org/10.6084/m9.figshare.5072782.v1).

The mitogenome (15,573 bp) is standard in size and organization: 13 protein-coding genes (PCGs), two rRNAs, 22 tRNAs and AT-rich region, with 23 genes on the J strand. It is very compact, with 11 intergenic regions (56 nucleotides altogether) and 16 gene overlaps (max. was 9 bp: *tRNA^Ph^*\*ND5*), six of which were tRNA\tRNA, six PCG\tRNA and four PCG\PCG. Apart from *Cox1*, which uses TCG as start codon, all other genes use standard ATN/TAN start/stop codons, respectively. A + T bias is very high (81%), particularly in the AT-rich region (90%). All these characteristics are common for Diptera (Gissi et al. 2008; Nelson et al. 2012; Ding et al. 2015). Gene order and sizes are relatively conserved among the 14 compared Calyptratae mitogenomes.

Phylogenetic analysis was conducted using MrBayes 3.1.2 (Ronquist and Huelsenbeck 2003). We have selected a subset of 13 mitogenomes belonging to the first higher taxonomic category, Calyptratae subsection, at least two (where available) from each represented family. Two basal Brachycera were outgroups (Ding et al. 2015). Analyses were performed on concatenated PCGs and RNAs, but six tRNAs (L, S, Q, M, I and E) were removed as they were not consistent (either absent or duplicated) in all sequences. Sequences were retrieved, concatenated and aligned as described before (Wen et al. 2017).

Although posterior probability (pp) values were very high (apart from one node, where pp = 40), Oestroidea and Muscoidea superfamilies were paraphyletic (Figure 1). The studied *M. ovinus* sequence was nested within the Oestridae clade (pp = 89), thus rendering the family paraphyletic. Paraphyly within Calyptratae is not a novel finding (Kutty et al. 2010; Nelson et al. 2012; Shan et al. 2015), but these results might also be artefacts caused by the compositional bias (extremely high A + T) of dipteran mitogenomic sequences, causing mutational saturation and distorting the phylogenetic signal (Cameron 2014). A larger number of Hippoboscoidea mitogenomes will have to be available to achieve a better phylogenetic resolution.

**Figure 1.**
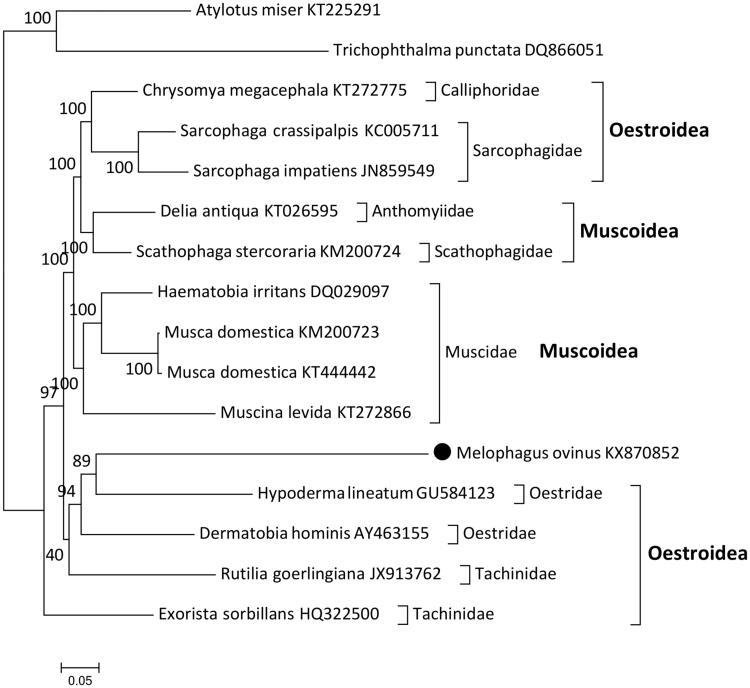
Phylogenetic tree inferred for the Calyptratae subsection. Phylogenetic analysis was conducted on 16 (14 Calyptratae + 2 outgroups) almost complete (PCGs + most of RNAs) mitogenomes using Bayesian inference (MrBayes), with default settings, 7 × 10^6^ generations and GTR + G + I evolution model. *Atylotus miser* and *Trichophthalma punctata* are outgroups. Bayesian posterior probability values are indicated next to nodes. *M. ovinus* sequence is highlighted by a black dot. Families and superfamilies (bold font) are indicated on the right. All sequences are shown with the corresponding GenBank accession numbers.
